# Cannabis and tolerance: acute drug impairment as a function of cannabis use history

**DOI:** 10.1038/srep26843

**Published:** 2016-05-26

**Authors:** J. G. Ramaekers, J. H. van Wel, D. B. Spronk, S. W. Toennes, K. P. C. Kuypers, E. L. Theunissen, R. J. Verkes

**Affiliations:** 1 Dept Neuropsychology and Psychopharmacology, Faculty of Psychology and Neuroscience, Maastricht University, Maastricht, The Netherlands; 2 Dept of Psychiatry, Donders Institute for Brain, Cognition and Behaviour, Radboud university medical center, Nijmegen, The Netherlands; 3 Dept of Forensic Toxicology, Institute of Legal Medicine, Goethe University of Frankfurt, Frankfurt, Germany; 4 The Netherlands Forensic Psychiatric Centre Pompestichting, Nijmegen, The Netherlands

## Abstract

Cannabis use history as predictor of neurocognitive response to cannabis intoxication remains subject to scientific and policy debates. The present study assessed the influence of cannabis on neurocognition in cannabis users whose cannabis use history ranged from infrequent to daily use. Drug users (N = 122) received acute doses of cannabis (300 μg/kg THC), cocaine HCl (300 mg) and placebo. Cocaine served as active control for demonstrating neurocognitive test sensitivity. Executive function, impulse control, attention, psychomotor function and subjective intoxication were significantly worse after cannabis administration relative to placebo. Cocaine improved psychomotor function and attention, impaired impulse control and increased feelings of intoxication. Acute effects of cannabis and cocaine on neurocognitive performance were similar across cannabis users irrespective of their cannabis use history. Absence of tolerance implies that that frequent cannabis use and intoxication can be expected to interfere with neurocognitive performance in many daily environments such as school, work or traffic.

Cannabis is the most widely used illicit drug in the world^1^. Population data suggests that 4% of the global population uses cannabis and that one out of ten users develops daily use patterns^2^. The prevalence of cannabis use is expected to increase following recent legalization of medical and recreational use in several countries worldwide and the introduction of a legal cannabis industry. Recent findings of the 2014 Monitoring the Future Survey funded by the National Institute of Drug Abuse indicated that in US states that have legalized cannabis, 40% of high school seniors had used cannabis compared with 26% in states that do not have legalized cannabis. Moreover, only 16.4% of high school seniors thought that cannabis smoking puts users at a greater risk^3^.

A solid volume of epidemiological and clinical research has established that cannabis use can produce adverse effects on cognitive function and mental health^4^ and increase risk of motor vehicle crashes^5^,^6^,^7^. Experimental, placebo controlled studies have repeatedly demonstrated that single doses of cannabis and THC cause a dose dependent reduction in performance as assessed with neurocognitive tasks measuring memory, attention, impulse control and motor function^8^,^9^,^10^,^11^,^12^,^13^. Performance impairments are maximal during the first hour after smoking, decline over 2–4 hrs after cannabis use^9^ and are detectable at serum ∆9-tetrahydrocannabinol (THC) concentrations as low as 2–5 ng/ml^14^.

The majority of experimental performance studies to date have been conducted in occasional users of cannabis with a low frequency of lifetime use. It has been suggested that performance impairment during cannabis intoxication is less in frequent cannabis users as a consequence of tolerance. Early studies of cannabis tolerance in humans suggest that prolonged administration of cannabis can reduce subjective^15^,^16^,^17^ and physiologic responses^18^ to cannabis intoxication. It is less clear however whether repeated use of cannabis will also alter the neurocognitive response because systematic studies are lacking. A few studies performed in small samples of heavy cannabis users (i.e. N = 12–24) indicated that cannabis produced minimal changes to cognitive and psychomotor function^19^,^20^,^21^,^22^,^23^. Other studies however indicated that heavy cannabis users remain very sensitive to the impairing effects of THC on impulse control^20^,^24^ or on a wider range of neurocognitive domains when assessed in a larger sample size^25^. Cannabis use history as predictor of neurocognitive response to cannabis intoxication therefore remains subject to scientific discussion as well as policy debates regarding cannabis-law reforms. For example, the potential for tolerance development has been raised as a practical concern against imposing per se laws for driving under the influence of cannabis^26^.

The present study was designed to systematically assess the influence of cannabis on performance in a large sample of cannabis users whose cannabis use history ranged from infrequent to daily use. Executive function, impulse control, attention and psychomotor function were assessed by means of a neurocognitive test battery with demonstrated sensitivity to cannabis intoxication^20^,^23^. Cannabis users received single doses of placebo, cannabis and cocaine on separate occasions. Cocaine served as an additional active control for demonstrating test sensitivity to drug challenges. It was expected that acute impairment following cannabis administration would be identical across users in the absence of tolerance and decrease as a function of cannabis use history in the presence of tolerance.

## Methods

### Participants

Participants were recruited at 2 study sites (i.e. Maastricht and Nijmegen) that participated in this multicenter trial. In total, 132 users of cannabis and cocaine (male N = 96, female N = 26) entered the study. Ten participants dropped out for various non-study related reasons and a total of 122 subjects completed all treatments conditions. Mean age of participants was 22.8 (min-max: 18–39) yrs. Participants were recruited through advertisements in local newspapers, flyers distributed at college campuses, bars, night clubs, concerts, head shops and by word of mouth. Candidates received a medical examination by the medical supervisors who determined study eligibility. The medical supervisor checked vital signs, conducted a resting 12-lead electrocardiogram (ECG), took blood and urine samples. Participants filled out a standard questionnaire on medical as well as drug use history. Standard blood chemistry, hematology and drug screen tests were conducted on blood and urine samples respectively. Inclusion criteria were: written informed consent; age 18–40 yrs; (regular) use of cannabis (i.e. ≥2 times/past 3 mo); cocaine use at least 5 times in the previous year, good physical and mental health and normal weight (BMI 18–28). Exclusion criteria were: cocaine dependence according to DSM-IV criteria; use of psychotropic medicinal drugs, presence or history of psychiatric or neurological disorder; pregnancy or lactating; cardiovascular abnormalities; excessive alcohol use (>20 units/week) or smoking (>15 cigarettes/day), and hypertension.

**Figure 1:**
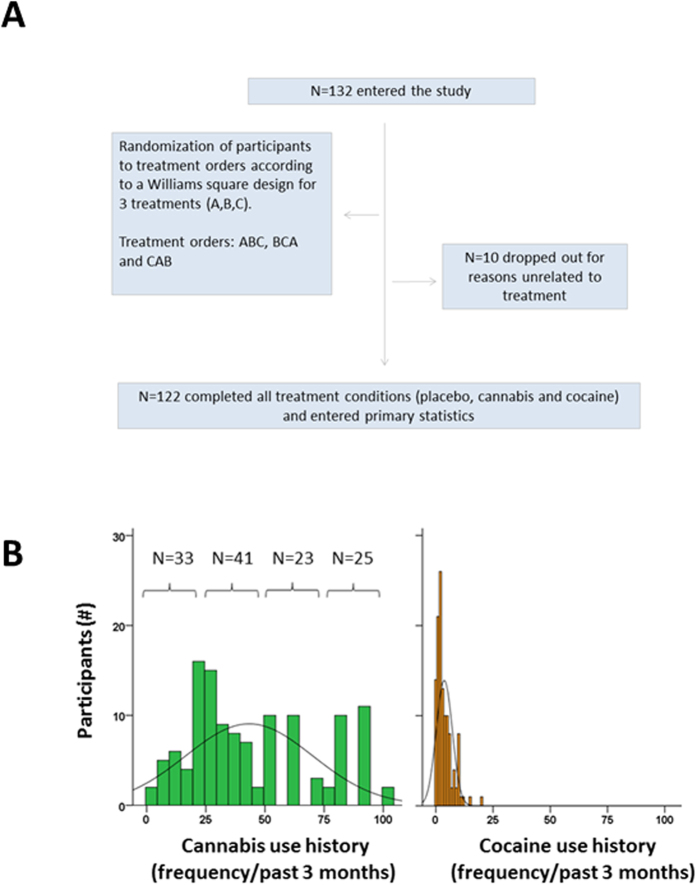
Randomization of participants (A) and frequency distribution of cannabis and cocaine use across study participants (N = 122) prior to study entrance and number of participants allocated to 4 cannabis use history groups (B).

On average, participants had been regular users of cannabis and cocaine for 7 yrs (min-max: 1–23) and 3.2 yrs (min-max: 0.5–6) respectively. They reported an average use of cannabis and cocaine on 44.8 and 3.7 occasions (i.e. a discreet event or period of subjective high caused by smoking one or more cigarettes) respectively during the previous 3 months. The frequency distribution of cannabis and cocaine use across study participants prior to study entrance is shown in Fig. 1. Frequency of cannabis use ranged between 2 and 100 occasions during 3 months prior to study entrance and included regular but infrequent users as well as daily users of cannabis. Participants were about equally distributed over the full range of cannabis use and were allocated to 4 mutually excluding cannabis use history groups: i.e. use on 1–24 (N = 33); 25–49 (N = 41), 50–74 (N = 23) and 75–100 (N = 25) occasions during the past 3 months. Frequency of cocaine use was relatively comparable across participants (range 1–20 times in the past 3 months) and did not differ between cannabis use history groups and consequently was not taken into account as a separate independent factor. Participants also reported the use of other substances such as MDMA (88%), amphetamines (73%), mushrooms (61%), LSD (20%) and a range of miscellaneous drugs (60%) such as nitrous oxide, DMT and ketamine.

This study was part of a large trial on the association between drug use, performance and impulse control (Dutch Trial Register, trial number NTR2127). The study was approved by the Medical Ethics Committee of Maastricht University and conducted according to the code of ethics on human experimentation established by the declaration of Helsinki (1964) and its amendments. Written informed consent was obtained from all subjects.

### Design, drug dose and administration

Participants entered a double-blind, placebo-controlled, 3-way crossover study. Treatments consisted of placebo, 300 μg/kg THC and a single dose of 300 mg cocaine HCl. Cannabis was prepared from batches containing 11–12% THC, a standard potency for cannabis sold at Dutch pharmacies. Cannabis placebo was prepared from a herbal plant mixture (Knaster) that contained no CNS active (i.e. THC) ingredients. Cannabis doses were tailored to each individual subject to represent weight calibrated doses of 300 μg/kg THC and administered using a Volcano vaporizer (Storz & Bickel GmbH & Co, Tuttlingen, Germany). Cannabis (placebo) was vaporized at a temperature of about 225 °C and the vapour was stored in a polythene bag equipped with a valved mouthpiece. Subjects were instructed to inhale deeply and hold their breath for 10 s after each inhalation. Within 2–3 min, the bag was to be fully emptied. Cocaine HCl and placebo were administered in an opaque white capsule. Treatments were administered using a double dummy technique to synchronize time of maximal drug concentrations (Tmax) during performance testing. Cocaine or cocaine placebo capsules were administered at 75 min prior (T0) to assessment of performance, whereas cannabis or cannabis placebo was inhaled 15 min prior (T1) to performance testing. Treatment conditions were separated by a minimum wash-out period of 7 days. Treatment orders were counterbalanced across participants.

### Procedures

Participants received training of tests and procedures on a separate day prior to the treatment conditions. Participants were instructed to refrain from drug use (except for cannabis) throughout the study. In the morning of test days, urine screens were used to check for the presence of benzodiazepines, opiates, cocaine, cannabis, MDMA and (meth)amphetamine. Subjects were also questioned about their last time of cannabis use upon arrival on test days. All subjects indicated that they did not smoke cannabis in the morning prior to testing. A breathalyzer was used to check for the presence of alcohol. Female participants underwent a pregnancy test. Participants were only allowed to proceed when test results for drug (except cannabis) and alcohol use and pregnancy were negative. Participants that passed the screen test received a standard breakfast, followed by baseline measures of vital signs (blood pressure and heart rate) and a sample of blood (prior to T0). Drug and placebo were administered according to the double dummy procedure explained above. Vital signs, blood samples and performance tests were scheduled within 5 min following T1. Performance tests were completed within 1 hr following T1.

### Neurocognitive performance

A neurocognitive test battery with demonstrated sensitivity to cannabis intoxication^20^,^23^,^25^ was employed to assess executive function, impulse control, attention and psychomotor function. The battery consisted of the tower of London (TOL), stop signal task (SST), critical tracking task (CTT), divided attention task (DAT) and a subjective evaluation of intoxication.

TOL is a decision-making task that measures executive function and planning^27^. It consists of computer-generated images of initial and target-arrangements of three colored balls on three sticks. The participant decides as quickly as possible, whether the end-arrangement can be accomplished in 2, 3, 4, or 5 steps from the initial arrangement by pushing the corresponding coded button. The total number of correct decisions is the main outcome measure.

SST measures motor impulsivity^28^, which is defined as the inability to inhibit an activated or pre-cued response leading to errors of commission. The task requires participants to make quick key responses to visual go signals (Go-trials), i.e. the letters ABCD presented one at a time in the middle of the screen, and to inhibit any response when a visual stop signal, i.e. “*” in one of the four corners of the screen, is presented at predefined delays (No-go trials). The major dependent variable is the number of commission errors on stop trials.

CTT measures the ability to control a displayed error signal in a first-order compensatory tracking task^29^. Error is displayed as a horizontal deviation of a cursor from the midpoint on a horizontal, linear scale. Compensatory joystick movements null the error by returning the cursor to the midpoint. The frequency at which the participant loses control is the critical frequency or lambda. The test includes five trials of which the lowest and the highest score are removed; the average of the remaining scores is taken as the final lambda-c (rad/sec) score.

DAT measures the ability to divide attention between two tasks performed simultaneously^20^. Participants were asked to perform the same tracking task as described above but now at a constant level of difficulty. As a secondary task, the participant was instructed to monitor 24 single digits (0 to 9) that were presented in the four corners of the computer screen (6 digits per corner). These numbers changed asynchronously every 5 seconds. The participants were instructed to react to the target number ‘2’ by removing their foot as fast as possible from a pedal switch and return. Average tracking error (mm), and percentage of correct responses (hits) to the target are the dependent performance measures.

Subjective intoxication was measured by means of a visual analogue scale (10 cm). Participants rated their level of ‘intoxication’, relative to their highest level of intoxication ever (0 = no intoxication, 10 = extremely intoxicated).

### Pharmacokinetics

Serum was used for detection of cannabinoids whereas cocaine and metabolites were determined in sodium fluoride stabilized plasma. The determination of THC, 11-hydroxy-THC (THC-OH), 11-nor-9-carboxy-THC (THC-COOH), cocaine (COC), benzoylecgonine (BZE) and ecgonine methyl ester (EME) in plasma was performed in a specialized forensic-toxicological laboratory using validated procedures^30^,^31^.

### Statistics

A total of 122 participants completed all treatment conditions (see Fig. 1a). Data was missing in a varying number of participants for each neurocognitive task . Only participants with complete datasets entered the statistical analyses for subjective intoxication (N = 120), TOL (N = 113), SST (N = 114), CTT (N = 113) and DAT (N = 92). The association between performance during drug intoxication and cannabis use was analyzed according to the following steps. First, a GLM repeated measures ANOVA with Drug (3 levels) as within subject factor and Cannabis use history (4 levels) as between group factor was conducted to establish overall effects of both factors and their interaction. The GLM model subsequently established the effects of cannabis and cocaine and their interaction with cannabis use history separately by means of drug-placebo contrasts. Second, difference scores from placebo were calculated for all participants and in all drug conditions. Individual changes (from placebo) in performance during cannabis and cocaine were subsequently correlated (Pearson-r) to individual frequencies of cannabis use over the past 3 months.

## Results

### Neurocognitive performance

**Figure 2:**
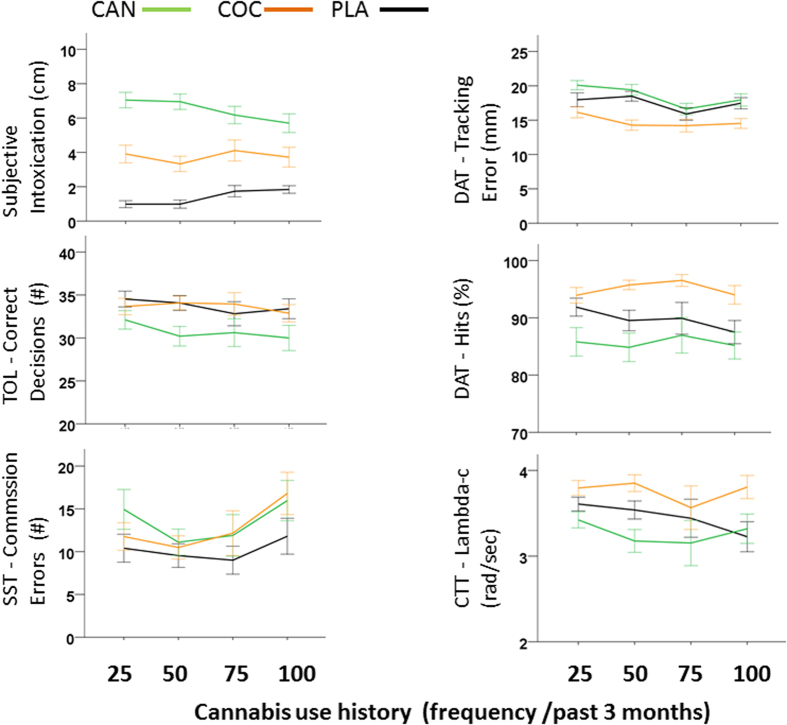
Mean (se) subjective intoxication and task performance following acute doses of cannabis (CAN), cocaine (COC) and placebo (PLA) as a function of cannabis use history.

Mean (se) subjective intoxication and task performance following acute doses of cannabis, cocaine and placebo as a function of cannabis use history are shown in Fig. 2. GLM analyses revealed significant (p < 0.05) overall effects of the factor Drug on every neurocognitive measure and significant overall interactions between the factor Drug and Cannabis use history on CTT (lambda-c) and DAT (tracking error) measures. There was no main effect of the factor Cannabis use history. Subsequent contrasts revealed that cannabis induced feelings of intoxication and produced impairment on all performance measures. Cocaine also induced feelings of intoxication, improved performance on CTT and DAT and impaired performance on SST. CTT performance during cannabis and cocaine intoxication significantly varied as a function of cannabis use history, indicating that cocaine-induced stimulation increased and cannabis-induced impairment decreased with increasing frequency of cannabis use. All other measures did not reveal any interaction between cannabis or cocaine intoxication and cannabis use history. Subjective intoxication following cannabis administration tended (p = 0.07) to decrease with increasing frequency of cannabis use. A summary of GLM drug-placebo contrasts for all measures is provided in Table 1.

**Figure 3:**
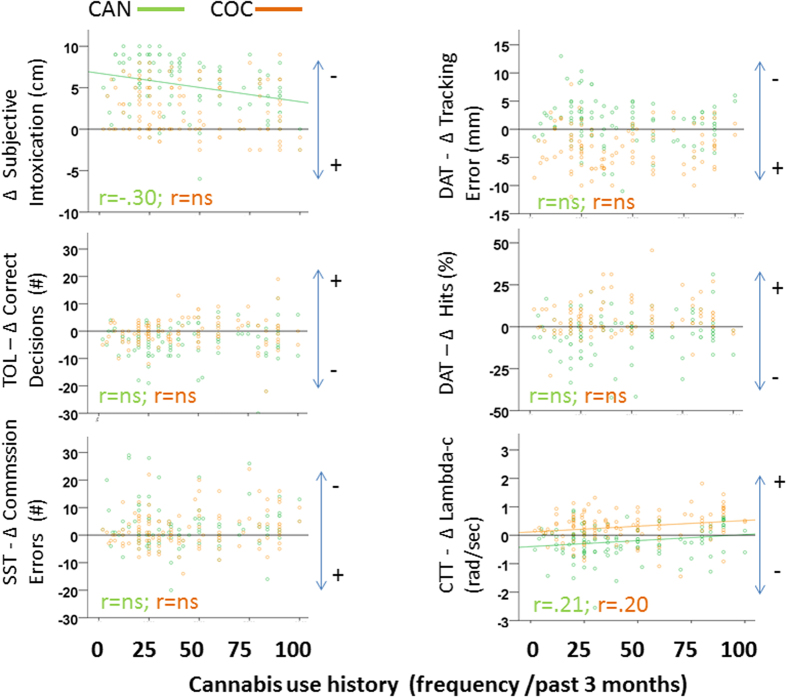
Scatterplots of individual changes (from placebo) in subjective intoxication and task performance following acute doses of cannabis (CAN) and cocaine (COC) as a function of cannabis use history. Significant (p < 0.05) correlations (r) between changes in subjective intoxication/task performance and cannabis use history are shown. (Impairment  = −; Improvement = +).

Correlational analyses between individual changes (from placebo) and individual cannabis use frequency confirmed GLM findings. Frequency of cannabis use correlated significantly with change in subjective intoxication following cannabis administration and with change in CTT performance following both cannabis and cocaine. A summary of (significant) correlations between individual performance changes during drug intoxication and cannabis use frequency is shown in Fig. 3.

### Control measures

One-way ANOVA revealed that THC and cocaine concentrations after administration of cannabis and cocaine did not significantly differ between cannabis use history groups. Mean concentrations of THC, cocaine and their main metabolites averaged across all participants are given in Table 2.

**Figure 4:**
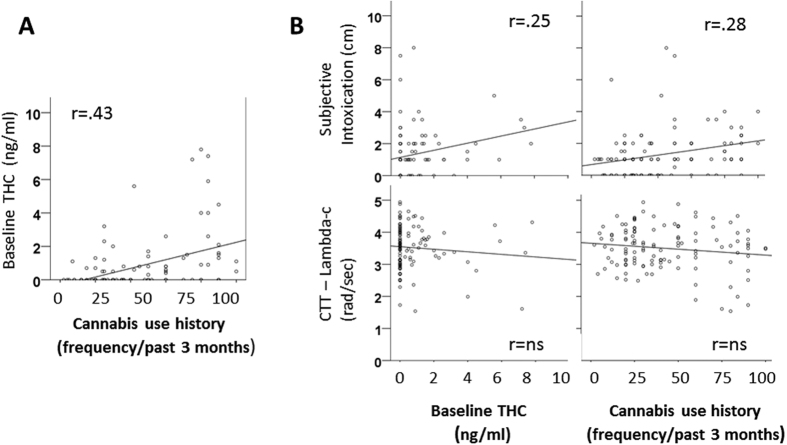
Scatterplots showing significant (p < 0.05) correlations (r) between (A) THC concentration at baseline and cannabis use history and between (B) CTT task performance/subjective intoxication and baseline THC/cannabis use history during placebo.

Mean baseline THC concentrations prior to drug and placebo administration did differ between cannabis use history groups (F_3,338_ = 21,7; p = 0.000) and were highest in (near) daily users. Baseline THC concentrations were significantly correlated with frequency of cannabis use (r = 0.38) across treatment conditions: i.e. higher concentrations were associated with more frequent use. Baseline THC and cannabis use history correlated significantly with subjective intoxication, but not CTT performance, during placebo. Baseline THC did not correlate with performance and subjective intoxication following cannabis and cocaine administration. A summary of significant correlations between baseline THC, cannabis use history, subjective intoxication and CTT performance in the placebo condition is shown in Fig. 4.

## Discussion

The current study confirmed the presence of performance impairment across a wide range of neurocognitive domains during cannabis intoxication. Executive function, impulse control, attention and psychomotor function were significantly worse after cannabis administration as compared to placebo. These findings are in line with earlier studies demonstrating acute cannabis impairment in the same performance domains^9^,^20^,^23^,^25^. Likewise, cocaine effects on neurocognitive performance were also in the expected directions. The drug improved psychomotor function and attention but impaired impulse control. Similar findings have been reported before^25^ and are in line with the well characterized CNS stimulant properties of cocaine^32^.

The present sample represented a diversity of cannabis users whose cannabis use history ranged from infrequent to daily use. Participants were about equally distributed over the full range of cannabis use which allowed for reliable assessment of acute drug effects in cannabis users with varying habits of use. In general, acute effects of cannabis and cocaine on neurocognitive performance were similar across cannabis users irrespective of their cannabis use history. Drug effects on executive function, impulse control and attention did not interact or correlate with cannabis use history. Two measures did indicate a significant contribution of cannabis use history to the neurocognitive response to drug intoxication. Frequency of cannabis use correlated significantly with change in subjective intoxication following cannabis administration and also correlated and interacted with (changes in) psychomotor performance (i.e. CTT) following both cannabis and cocaine. This suggests that subjective intoxication and psychomotor impairment following cannabis administration decrease with increasing frequency of cannabis use and that the stimulatory effect of cocaine on psychomotor function increases with increasing frequency of cannabis use.

The current interactions between drug effects on subjective intoxication and psychomotor function and cannabis use history should however be interpreted with caution. Inspection of the data shows that these were mainly caused by performance fluctuations during placebo rather than during drug conditions. Psychomotor performance during placebo declined with increased cannabis use while performance during cannabis and cocaine was relatively stable. Likewise, subjective intoxication ratings during placebo were slightly higher in frequent users as compared to infrequent cannabis users. Differences in subjective intoxication and psychomotor function between infrequent and frequent cannabis users during placebo may represent differences in baseline THC concentration across participants. Many participants were regular cannabis users who tested positive for THC prior to drug and placebo administration. Baseline THC levels indicate accumulated residuals from repeated use or recent use. The presence of low levels of THC in frequent users may have caused some degree of subjective high and performance impairment during placebo treatments.

Baseline THC levels were positively correlated to cannabis use history and generally higher in frequent cannabis users. Confounding between baseline THC and frequency of cannabis use was also evident from the finding that both factors were significantly correlated to subjective intoxication during placebo. Psychomotor performance during placebo did not correlate with baseline THC and cannabis use history which is in line with previous studies showing that associations between THC concentrations and performance impairment are generally weak or absent^6^,^14^. Yet, baseline THC levels were high enough to expect intoxication and mild psychomotor impairment in some individuals^14^. Psychomotor function as measured by CTT has also been shown to be more sensitive to low levels of THC (i.e. between 2–5 ng/ml) as compared to other performance measures such as TOL and SST^14^, which may explain why cannabis use history interacted with (placebo) performance in some measures but not in others.

Overall, the present study demonstrates that cannabis induced impairment does not depend on cannabis use history and indicates that tolerance to impairing effects of cannabis on neurocognitive function is generally absent in frequent users. These data confirm previous suspicions that neurocognitive impairments during cannabis intoxication do occur in infrequent as well as frequent cannabis users^20^,^25^,^33^,^34^. Previous studies reporting absence of neurocognitive impairment during cannabis intoxication in heavy, daily users employed small samples sizes^19^,^20^,^23^,^35^,^36^ or failed to measure and control for baseline THC^19^,^21^ which may have decreased study sensitivity. Alternatively, cannabis use frequency of participants is difficult to compare between studies but was estimated to be about twice as high in some studies^19^,^21^ as compared to those of (near) daily users in the present study. This suggests that tolerance to cannabis impairment may develop far beyond cannabis use frequencies reported in this paper. Still, the current study presents an integrative approach that employed a large sample ranging from infrequent to daily cannabis users to address the issue of cannabis intoxication and tolerance while controlling for THC concentration in blood. The present demonstration of absence of tolerance to cannabis impairment has important implications for risk perception in frequent users. It implies that neurocognitive function of daily or near daily cannabis users can be substantially impaired from repeated cannabis use, during and beyond the initial phase of intoxication. As a consequence, frequent cannabis use and intoxication can be expected to interfere with neurocognitive performance in many daily environments such as school, work or traffic.

## Additional Information

**How to cite this article**: Ramaekers, J. G. *et al.* Cannabis and tolerance: acute drug impairment as a function of cannabis use history. *Sci. Rep.* **6**, 26843; doi: 10.1038/srep26843 (2016).

## Figures and Tables

**Table 1:** Summary of significant changes (p, F) in subjective intoxication and performance measures induced by cannabis (CAN), cocaine (COC) and their interaction with Cannabis Use History (CUH) as indicated by GLM drug-placebo contrast analyses.

	CAN	CAN × CUH	COC	COC × CUH
Subjective intoxication	0.000, F_1,116_ = 357,6	–	0.000, F_1,116_ = 75,6	–
TOL - correct decisions	0.000, F_1,109_ = 27,6	–	–	–
SST - commission errors	0.000, F_1,110_ = 15,5	–	0.000, F_1,110_ = 15,1	–
DAT - tracking error	0.019, F_1,88_ = 5,7	–	0.000, F_1,88_ = 55,7	–
DAT – hits	0.010, F_1,88_ = 6,9	–	0.000, F_1,88_ = 28,6	–
CTT - lambda-c	0.000, F_1,109_ = 13,9	0.032, F_3,109_ = 3.1	0.000, F_1,109_ = 39,7	0.008, F_3,109_ = 4.2

**Table 2:** Mean (sd) concentrations of THC, cocaine and their main metabolites at baseline and after drug administration (i.e. 5 min post smoking cannabis and 65 min post oral cocaine).

	THC [ng/ml]	THC-OH [ng/ml]	THC-COOH [ng/ml]	Cocaine [mg/L]	BZE [mg/L]	EME [mg/L]
*Baseline*	1.57 (4.14)	0.62 (1.72)	20.91 (37.68)	0.00 (0.0)	0.00 (0.00)	0.00 (0.00)
*Post drug*	73.81 (63.09)	6.86 (4.32)	38.69 (33.38)	0.25 (0.19)	0.49 (0.27)	0.14 (0.11)
