# Erratum: Cannabis and tolerance: acute drug impairment as a function of cannabis use history

**DOI:** 10.1038/srep31939

**Published:** 2016-09-02

**Authors:** J. G. Ramaekers, J. H. van Wel, D. B. Spronk, S. W. Toennes, K. P. C. Kuypers, E. L. Theunissen, R. J. Verkes

Scientific Reports 6: Article number: 2684310.1038/srep26843; published online: 05 26 2016; updated: 09 02 2016

This Article contains an error in the Author Contributions statement.

“J.G.R. and R.J.V. designed the study and operated as PIs. J.H.v.W., D.B.S., K.P.C.K. and E.L.T. were responsible for study management. J.H.v.W. and D.B.S. were responsible for collection and processing of research data. S.W.T. was responsible for the analysis of PK data. R.J.V. conducted statistical analysis and wrote the paper. All authors reviewed the paper”.

should read:

“J.G.R. and R.J.V. designed the study and operated as PIs. J.H.v.W., D.B.S., K.P.C.K. and E.L.T. were responsible for study management. J.H.v.W. and D.B.S. were responsible for collection and processing of research data. S.W.T. was responsible for the analysis of PK data. J.G.R. conducted statistical analysis and wrote the paper. All authors reviewed the paper”.

